# Differential Association of Dietary Linoleic Acid and Alpha-linolenic Acid with Adipose Tissue in a Sample of Iranian Adults; A Cohort-based Cross-sectional Study

**DOI:** 10.31661/gmj.v12i.3023

**Published:** 2023-07-29

**Authors:** Esmail Karami, Saeid Hadi, Mohsen Mohit, Seyed Jalil Masoumi

**Affiliations:** ^1^ Lifestyle Institute, Baqiyatallah University of Medical Sciences, Tehran, Iran; ^2^ Department of Nutrition, School of Medicine, AJA University of Medical Sciences, Tehran, Iran; ^3^ Student Research Committee, Department of Clinical Nutrition, School of Nutrition and Food Sciences, Shiraz University of Medical Sciences, Shiraz, Iran; ^4^ Department of Clinical Nutrition, School of Nutrition and Food Sciences, Shiraz University of Medical Sciences, Shiraz, Iran

**Keywords:** Fatty acids, Linoleic Acids, Alpha-linolenic Acid, Body Fat, Overweight

## Abstract

Background: Overweight and obesity are the most critical risk factors for chronic diseases. The quality of dietary fatty acids as one of the factors affecting fat accumulation has received little attention. This study investigates the association between dietary linoleic acid (LA) and alpha-linolenic acid (ALA) with body fat indices in a sample of healthy Iranian adults.Materials and Methods: In this cohort-based cross-sectional study, 3,195 individuals aged 20 to 60 who participated in the Shiraz University of Medical Science Employees Health Cohort study were included. Dietary intake was assessed using a validated 118-item Food Frequency Questionnaire (FFQ), and body composition was assessed by the bioelectrical impedance analysis method. Multiple linear regression adjusted for relevant confounders was used to determine the associations.Results: Mean dietary intake of LA was 14.20 ± 7.01 mg/day for men and 13.90 ± 6.71 mg/day for women. Additionally, the daily intake of ALA was 0.18 ± 0.18 mg/day in men and 0.17 ± 0.19 mg/day in women. Dietary intake of ALA for men had an inversely significant association with body fat mass (BFM) (β: -0.585, 95% CI: -1.137, -0.032, P=0.038), percentage of body fat (PBF) (β: -0.537, 95% CI: -0.945, -0.129, P=0.010), Visceral Fat Area (VFA) (β: -2.998, 95% CI: -5.695, -0.302, P=0.029), and Waist to Hip Ratio (WHR) (β: -0.689, 95% CI: -1.339, -0.040, P=0.038).Conclusion: Higher dietary ALA intake was associated with lower BFM, BFP, VAF, and WHR in men. The present study confirms that ALA intake should be considered a preventive treatment to improve body composition. However, further research is recommended in this regard.

## Introduction

Overweight and obesity have doubled during the last 40 years. About 30% of the world’s population faces abnormal body fat accumulation [1]. The overweight prevalence among Iranian adults reported 35.8% (37% men, 35% women), 22.3% (16% men, 26.3% women) for obesity prevalence, and 31.1% (15.6% men, 41.2% women) for central obesity prevalence [2]. Body fat is a mixture of essential and storage fat. Essential fats are found in small amounts in bone marrow, heart, lung, liver, kidney, and nervous system; in men, about 3% of body fat is essential, and in women, this amount is 12% of body fat. Storage fat in adipose tissue is mainly in the form of triglycerides, which are under the skin and internal organs to protect them against damage. The amount of total body fat that is related to health is 18-24% in men and 31-25% in women [3]. Excess body fat and visceral fat area (VFA) is strongly associated with adverse metabolic outcomes, including a disturbed lipid profile, blood glucose imbalance, and insulin resistance [4][5]. This condition will increase the chances of cardiovascular diseases, cancers, and diabetes [6][7]. Several approaches including surgery, pharmacological therapy, and diet therapy have been proposed for obesity treatment [8].

Dietary factors play a key role in obesity management [9]. Although the intercellular pathways that influence the distribution of body fat mass are not well defined, dietary components and macronutrient composition can lead to different body fat distribution patterns [10][11]. Among the macronutrients, fats are more accused of adipose tissue accumulation because they produce more energy than carbohydrates and proteins by providing nine kcal/g [12].

The percentage of dietary fat in the total dietary energy is estimated to be 20-35% to prevent chronic diseases [13]. A direct association was found between excess dietary fat and body fat mass [14]. Fatty acids have different effects on body composition and fat distribution, but there is no consensus on the exact impact of each [15][16]. Also, it has been confirmed that dietary fatty acid composition affects obesity-related genes and is correlated with some mutations [17]. Polyunsaturated Fatty Acids (PUFA) have at least two double bonds in their chemical structure [18]. PUFAs with more than 20 carbons are called long-chain polyunsaturated fatty acids (LC-PUFA) [18]. Based on therapeutic lifestyle change (TLC) recommendations, a maximum of 10% of total energy intake should be allocated to PUFAs [19]. Linoleic acid (LA) and alpha-linolenic acid (ALA) are essential LC-PUFAs with double bonds in the sixth (Omega-6) and third bonds (Omega-3) from the methyl side, respectively. LA and ALA are used to produce some other essential fatty acids in the body [20][21]. Insufficient intake of LA and ALA, causes deficiency symptoms. It is recommended to provide 1-2% of an individual’s energy intake by LA and ALA, separately [22]. The main dietary sources of LA include soybean oil, corn oil, sunflower oil, and almond oil [23]. Also, the main dietary sources of ALA include canola oil, flaxseed oil, fish oil, and chia seeds [20].

To date, there was no definitive consensus on the relation between specific PUFAs, including ALA and LA, and adipose tissue [24][25][26][27]. This study aimed to investigate the association between dietary LA and ALA with body fat mass (BFM), percentage of body fat (PBF), VFA, body mass index (BMI), waist circumference (WC), hip circumference (HC), and waist-to-hip ratio (WHR) in a sample of Iranian adults.

## Materials and Methods

Ethics

This study was approved by the Ethics Committee of Shiraz University of Medical Sciences (No: IR.SUMS.REC.1399.744). Also, all the methods of the current research were performed according to the Helsinki guidelines [28]. Informed consent was obtained from all the participants.

Study Population

The current study is a cohort-based cross-sectional study conducted on the baseline data obtained from August 2017 to February 2020 that was obtained from the Employees Health Cohort registry system of the Shiraz University of Medical Science. The subjects were employees of Shiraz University of Medical Sciences and aged between 20-60 years old. Individuals with chronic diseases including high blood pressure, diabetes, and cardiovascular disease unable to communicate to answer (blind, deaf, dumb, and paralyzed people unable to travel to the cohort center or patients with mental and psychological disorders) were also excluded from the study.

Data Collection

Participants’ demographic data including gender (male, female), age (years), marital status (single, married, widow, and divorced), recent educational degree (less than bachelor’s degree, bachelor’s degree or higher), number of children (under three, three and higher) were collected via a standard social-demographic questionnaire. Also, the mobility and physical activity of subjects was assessed using the international physical activity questionnaire (IPAQ) [29]. Physical activity scores were calculated for walking (3/3 MET- min/week), moderate (4 MET- min/week), and vigorous activity (8 MET- min/week).

Dietary Assessment

The dietary intake was assessed using a reliable and validated 118-item food frequency questionnaire (FFQ) through face-to-face interviews with the participants. The validity of energy and nutrient estimates using FFQ has been confirmed among Iranian adults [30]. FFQ consists of standard serving sizes of foods. Data was collected on daily, weekly, monthly, and annual consumption of each food. FFQs were completed by an experienced nutritionist. The amount of consumed food was converted to grams of food per day, and then intake of energy and nutrient were obtained using the Nutritionist IV software (version 4.0, supplied by First Databank, San Francisco, United States).

Anthropometric Assessment

Participants’ height was measured with an accuracy of 0.5 cm using a wall stadiometer after standing without shoes (Seca, Germany). Also, the weight was recorded by a scale with an accuracy of 100 g (Seca808; Seca, Germany) with at least clothes. WC was measured in the middle of the distance between the lowest rib margin and the iliac crest with a tape measure while exhaling and standing. HC was measured with tape around the widest part of the hip over light clothes. All of the measurements were evaluated by trained experts. WHR was obtained by dividing WC (cm) by HC (cm). BMI was also calculated by dividing weight (Kg) by the square of the height (m2) [31].

The bioelectric impedance analysis (BIA) method and body composition measurement device (InBody 770, InBody BSM170; made in South Korea) was used to evaluate body fat. After the calibration of the device, participants stood on the device in their normal attire, without shoes. They held the pads at a 45-degree angle so that the device could perform body analysis.

Statistical Analysis

Quantitative and qualitative data are presented as mean ± standard deviation (SD) and frequency (percent), respectively. Because the total energy intake plays a determining role in micronutrient intake, we adjusted the dietary intake of LA, and ALA for total energy intake and then categorized them into tertiles. The residual method was employed for adjusting the dietary LA and ALA from total energy intake. One-way analysis of variance (ANOVA) and the chi-squared test were used to compare the means of quantitative and categorical variables across tertiles of adjusted dietary LA and ALA in both genders. The association between dietary LA and ALA and body fat parameters was investigated using multiple linear regression with a 95% confidence interval. SPSS version 26 (developed by IBM, Chicago, United States) software was used to perform the analyses. The P<0.05 is considered a significant level.

## Results

In the current cross-sectional study, out of 3195 subjects, men contained 43.91% (n=1403) participants with a mean age of 40.89 ± 7.23 years, and women included 56.09 % (n=1792) participants with a mean age of 40.99 ± 6.76 years. Total energy intake (2440.89 ± 772.84, P:<0.001), dietary LA intake (14.58 ± 7.36, P=0.007), and dietary ALA intake (0.20 ± 0.18, P<0.001) significantly higher in men than in women.

Other demographic characteristics of study subjects, categorized by gender, and the tertiles of energy-adjusted intake of LA and ALA are presented in Tables-1 and -2.

**Table:** Table1. Demographic Characteristics of Study Subjects by Gender

Variables	Total (n=3195)	Women (n=1792)	Men (n=1403)	p-value
Age (years)	40.95 ± 6.97	40.99 ± 6.76	40.89 ± 7.23	0.695
Marital Status, n (%) Single Married Other (divorced, widow)	457 (14.30%) 2580 (80.75%) 158 (4.94%)	344 (19.2%) 1309 (73.04%) 139 (7.75%)	113 (8.05%) 1271 (90.60%) 19 (1.35%)	<0.001
Last education degree, n (%) Under BSc BSc and higher	1176 (36.80%) 2019 (63.20%)	503 (28.34%) 1289 (71.93%)	673 (47.96%) 730 (52.03%)	<0.001
Number of children, n (%) Under 3 3 and higher	2868 (89.76%) 327 (10.23%)	1658 (92.52%) 135 (7.53%)	1210 (86.24%) 192 (13.68%)	<0.001
Physical activity (Met-min/week) Low and Moderate High	1892 (59.21%) 1303 (40.79%)	1160 (64.73%) 632 (35.26%)	732 (52.17%) 671 (47.82%)	<0.001
Energy intake (Kcal/d)	2172.47 ± 728.89	1962.46 ± 615.97	2440.89 ± 772.84	<0.001
Dietary LA intake (g/d)	14.20 ± 7.01	13.90 ± 6.71	14.58 ± 7.36	0.007
Dietary ALA intake (g/d)	0.18 ± 0.18	0.17 ± 0.19	0.20 ± 0.18	<0.001

Quantitative data are presented as mean ± standard deviation, qualitative data are presented as number (%). The p-value was obtained from a one-way analysis of variance test (ANOVA) and chi-square test for quantitative and qualitative data, respectively. A significant P-value is considered at P<0.05.

The crude and multifactorial adjusted coefficients (β) with 95 percent confidence intervals of body fat indicators across tertiles of dietary intake of LA and ALA in women and men are shown in Tables-3 and -4, respectively. Additionally, no significant association was observed between tertiles of dietary intake of LA and body fat parameters in both males and females. There was a significant inverse association in the second adjusted model for BFM, PBF, VFA, and WHR in men, in which a higher dietary ALA intake was associated with a lower BFM (β: -0.585, 95% CI: -1.137, -0.032, P=0.038), PBF (β: -0.537, 95% CI: -0.945, -0.129, P=0.010), VFA (β: -2.998, 95% CI: -5.695, -0.302, P=0.029), and WHR (β: -0.689, 95% CI: -1.339, -0.040, P=0.038). Also, unlike men, in women, no significant association was found between dietary ALA intake and body fat in different models (Table-3).

## Discussion

**Table:** **Table 2** Demographic Characteristics of Study Subjects by Energy-adjusted Tertiles of LA and ALA Intake

**Variables**			**LA**				**ALA**	
	**T1** **(n=1065)**	**T2** **(n=1065)**	**T3** **(n=1065)**	**p-value**	**T1** **(n=1065)**	**T2** **(n=1065)**	**T3** **(n=1065)**	**p-value**
**Age (years)**	41.78 ± 7.17	40.99 ± 6.83	40.08 ± 6.82	<0.001	41.47 ± 6.91	41.25 ± 6.94	40.12 ± 7.01	<0.001
**Gender, n (%)** Male Female	585 (54.92%) 480 (45.08%)	425 (39.90%) 640 (60.10%)	393 (36.90%) 672 (63.10%)	<0.001	600 (56.33%) 465 (43.66%)	391 (36.71%) 674 (63.29%)	412 (38.66%) 653 (61.33%)	<0.001
**Marital Status, n (%)** **Single** Married Other (divorced, widow)	134 (12.58%) 890 (83.56%) 41 (3.86%)	154 (14.50%) 857 (80.50%) 54 (5%)	169 (15.87%) 833 (78.22%) 63 (5.91%)	0.031	146 (13.70%) 880 (82.63%) 39 (3.67%)	170 (15.96%) 830 (77.93%) 65 (6.10%)	141 (13.3%) 870 (81.7%) 54 (5%)	0.024
**Last education degree, n (%)** Under BSc BSc and higher	393 (36.90%) 672 (63.10%)	382 (35.86%) 683 (64.13%)	401 (37.65%) 664 (62.35)	0.693	461 (43.28%) 604 (56.72%)	354 (33.23%) 711 (66.77%)	361 (33.88%) 704 (66.11%)	<0.001
**Number of children, n (%)** Under 3 3 and higher	923 (86.66%) 142 (13.33%)	877 (82.35%) 188 (17.65%)	971 (91.17%) 94 (8.83%)	<0.001	921 (86.47%) 144 (13.52%)	965 (90.61%) 100 (9.39%)	974 (91.45%) 91 (8.55%)	<0.001
**Physical activity (Met-min/week)** Low and Moderate High	659 (61.87%) 406 (38.12%)	633 (59.43%) 432 (40.57%)	600 (56.33%) 465 (43.66%)	0.33	623 (58.50%) 442 (41.50%)	675 (63.38%) 390 (36.62%)	594 (55.77%) 471 (44.22%)	0.001
**Energy intake** (Kcal/d)	2194.80 ± 824.19	2060.88 ± 638.09	2261 ± 698.19	<0.001	2386.10 ± 844.18	1938.51 ± 562.16	2192 ± 682.13	<0.001
**Total LA intake** (g/d)	8.73 ± 2.85	12.72 ± 3.32	21.15 ± 6.93	<0.001	14.09 ± 6.88	12.50 ± 5.73	16.00 ± 7.83	<0.001
**Total ALA intake** (g/d)	0.14 ± 0.09	0.17 ± 0.11	0.24 ± 0.28	<0.001	0.10 ± 0.05	0.13 ± 0.05	0.32 ± 0.26	<0.001

Quantitative data are reported as mean ± standard deviation, qualitative data are presented as number (%). The P-value was obtained from a one-way analysis of variance test (ANOVA) and chi-square test for quantitative and qualitative data, respectively. The significant P-value is considered at P<0.05.**LA;** linoleic acid,** ALA;**alpha-linolenic acid

**Table:** Table3. Association of Dietary LA and ALA Intake with Body Fat and Anthropometric Indices in Iranian Women.

**Variables**				**LA**				**ALA**	
**Men**		**β ± SE**	**R ^2^**	**95 % CI**	**p-value**	**β ± SE**	**R ^2^**	**95 % CI**	**p-value**
BFM	Crude	0.068 ± 0.230	<0.001	-0.383, 0.520	0.767	0.126 ± 0.234	<0.001	-0.333, 0.584	0.591
	Model 1	0.130 ± 0.226	0.067	-0.312, 0.572	0.565	0.284 ± 0.229	0.06	-0.164, 0.733	0.214	
	Model 2	0.099 ± 0.225	0.076	-0.342, 0.541	0.659	0.326 ± 0.229	0.077	-0.123, 0.775	0.155
PBF	Crude	0.096 ± 0.172	<0.001	-0.242, 0.434	0.577	-0.176 ± 0.175	0.001	-0.520, 0.167	0.313
	Model 1	0.150 ± 0.168	0.070	-0.181, 0.480	0.375	-0.037 ± 0.171	0.069	-0.372, 0.298	0.828
	Model 2	0.145 ± 0.169	0.072	-0.186, 0.476	0.390	-0.032 ± 0.172	0.071	-0.369, 0.305	0.851
VFA	Crude	0.651 ± 1.234	<0.001	-1.769, 3.071	0.598	-0.077 ± 1.253	<0.001	-2.533, 2.380	0.951
	Model 1	1.118 ± 1.199	0.081	-1.233, 3.468	0.351	0.932 ± 1.215	0.08	-1.152, 3.315	0.443
	Model 2	1.008 ± 1.199	0.086	-1.344, 3.361	0.401	1.081 ± 1.220	0.086	-1.311, 3.472	0.376
BMI	Crude	-0.028 ± 0.125	<0.001	-0.272, 0.217	0.825	0.035 ± 0.126	<0.001	-0.213, 0.283	0.782
	Model 1	0.009 ± 0.120	0.092	-0.227, 0.245	0.942	0.123 ± 0.122	0.092	-0.116, 0.362	0.313
	Model 2	-0.006 ± 0.120	0.104	-0.241, 0.229	0.960	0.141 ± 0.122	0.105	-0.098, 0.381	0.246
WC	Crude	-0.282 ± 0.295	0.001	-0.862, 0.297	0.339	-0.070 ± 0.300	<0.001	-0.658, 0.518	0.815
	Model 1	-0.129 ± 0.283	0.103	-0.685, 0.427	0.649	0.196 ± 0.287	0.103	-0.367, 0.760	0.494
	Model 2	-0.187 ± 0.283	0.111	-0.742, 0.368	0.509	0.273 ± 0.288	0.111	-0.292, 0.837	0.343
HC	Crude	-0.002 ± 0.229	<0.001	-0.451, 0.447	0.992	0.227 ± 0.232	0.001	-0.228, 0.683	0.328
	Model 1	0.045 ± 0.227	0.036	-0.401, 0.491	0.843	0.312 ± 0.230	0.037	-0.140, 0.764	0.176
	Model 2	-0.005 ± 0.227	0.047	-0.450, 0.440	0.981	0.381 ± 0.231	0.048	-0.071, 0.833	0.098
WHR	Crude	-0.003 ± 0.002	0.001	-0.007, 0.001	0.157	-0.003 ± 0.002	0.001	-0.007, 0.001	0.131
	Model 1	-0.002 ± 0.002	0.087	-0.005, 0.002	0.384	-0.001 ± 0.002	0.087	-0.005, 0.003	0.560
	Model 2	-0.002 ± 0.002	0.088	-0.006, 0.002	0.348	-0.001 ± 0.002	0.087	-0.005, 0.003	0.615

Results obtained from multiple linear regression analysis. Data presented as β coefficients (95%) ± standard error. **Model 1:** adjusted for age, Marital Status, Last education degree, number of children, and physical activity. **Model 2:** additionally, adjusted for energy intake, and dietary fiber. The significant P-value is considered at P<0.05.**LA;** linoleic acid,**ALA;**alpha-linolenic acid,**SE**; standard error, **BFM**; body fat mass, **PBF**; percentage of body fat, **VFA**; visceral fat area, **BMI**; body mass index, **WC**; waist circumference, **HC**; hip circumference, **WHR**; waist to hip ratio

**Table:** **Table- 4** Association of Dietary LA and ALA Intake with Body Fat and Anthropometric Indices in Iranian Women.

**Variables**				**LA**				**ALA**	
**Men**		**β ± SE**	**R ^2^**	**95 % CI**	**p-value**	**β ± SE**	**R ^2^**	**95 % CI**	**p-value**
**BFM**	Crude	0.112 ± 0.286	<0.001	-0.449, 0.673	0.696	-0.502 ± 0.281	0.002	-1.052, 0.049	0.074
	Model 1	0.140 ± 0.288	0.014	-0.425, 0.704	0.628	-0.569 ± 0.282	0.017	-1.123, -0.016	0.044
	Model 2	0.020 ± 0.287	0.032	-0.543, 0.582	0.945	-0.585 ± 0.282	0.035	-1.137, -0.032	***0.038***
**PBF**	Crude	-0.233 ± 0.211	0.001	-0.648, 0.181	0.269	-0.559 ± 0.207	0.005	-0.964, -0.153	0.007
	Model 1	-0.114 ± 0.212	0.021	-0.530, 0.301	0.590	-0.504 ± 0.207	0.025	-0.911, -0.097	0.015
	Model 2	-0.162 ± 0.212	0.029	-0.578, 0.254	0.444	-0.537 ± 0.208	0.033	-0.945, -0.129	***0.010***
**VFA**	Crude	0.566 ± 1.395	<0.001	-2.171, 3.304	0.685	-2.744 ± 1.368	0.003	-5.428, -0.059	0.045
	Model 1	0.835 ± 1.406	0.012	-1.922, 3.593	0.552	-2.897 ± 1.378	0.015	-5.599, -0.195	0.036
	Model 2	0.258 ± 1.400	0.029	-2.488, 3.004	0.854	-2.998 ± 1.374	0.033	-5.695, -0.302	***0.029***
**BMI**	Crude	0.033 ± 0.134	<0.001	-0.230, 0.295	0.808	- 0.182 ± 0.131	0.001	-0.440, 0.075	0.165
	Model 1	0.027 ± 0.135	0.011	-0.237, 0.292	0.839	-0.225 ± 0.132	0.013	-0.485, 0.034	0.088
	Model 2	-0.038 ± 0.134	0.033	-0.301, 0.224	0.774	-0.225 ± 0.132	0.035	-0.482, 0.033	0.088
**WC**	Crude	0.397 ± 0.337	0.001	-0.263, 1.058	0.238	-0.582 ± 0.330	0.002	-1.230, 0.066	0.078
	Model 1	0.431 ± 0.339	0.014	-0.233, 1.096	0.203	-0.670 ± 0.332	0.016	-1.322, -0.018	0.044
	Model 2	0.283 ± 0.337	0.034	-0.378, 0.945	0.401	-0.689 ± 0.331	0.036	-1.339, -0.040	***0.038***
**HC**	Crude	0.400 ± 0.249	0.002	-0.088, 0.888	0.108	-0.203 ± 0.244	<0.001	-0.683, 0.276	0.406
	Model 1	0.321 ± 0.249	0.024	-0.168, 0.810	0.198	-0.378 ± 0.245	0.024	-0.858, 0.102	0.123
	Model 2	0.209 ± 0.248	0.041	-0.278, 0.696	0.400	-0.364 ± 0.244	0.042	-0.843, 0.114	0.136
**WHR**	Crude	0.000 ± 0.002	<0.001	-0.003, 0.003	0.883	-0.004 ± 0.002	0.004	-0.007, -0.001	0.016
	Model 1	0.001 ± 0.002	0.030	-0.002, 0.005	0.424	-0.003 ± 0.002	0.033	-0.006, 0.000	0.052
	Model 2	0.001 ± 0.002	0.039	-0.002, 0.004	0.573	-0.003 ± 0.002	0.042	-0.007, 0.000	***0.034***

Results obtained from multiple linear regression analysis. Data presented as β coefficients (95%) ± standard error. **Model 1:** adjusted for age, Marital Status, Last education degree, number of children, and physical activity. **Model 2:** additionally, adjusted for energy intake, and dietary fiber. The significant P-value is considered at P<0.05.**LA;** linoleic acid,**ALA;**alpha-linolenic acid,**SE**; standard error, **BFM**; body fat mass, **PBF**; percentage of body fat, **VFA**; visceral fat area, **BMI**; body mass index, **WC**; waist circumference, **HC**; hip circumference, **WHR**; waist to hip ratio

To the best of our knowledge, this was the first cohort-based cross-sectional study investigating the association of specific PUFAs, including dietary LA and ALA intake, with adipose tissue. Our finding showed that dietary intake of ALA in men had an inversely significant association with BFM, PBF, VFA, WC, and WHR.

Obesity has been an ongoing trend in many societies for decades[32]. The most effective approach to treat obesity is lifestyle changes including a restrictive diet and increasing physical activity.

However, weight loss diets recommend reducing the percentage of fats, and all fatty acid types were reduced with this approach. Dietary fatty acid quality has received little attention in weight loss diets. Our study indicated that dietary AL and ALA can have different effects on adipose tissue, so it is recommended to reduce the dietary sources of LA and increase the dietary sources of ALA instead of reducing the dietary sources of both fatty acids in weight loss diets.

Consistent with these results, a clinical trial reported that consuming ALA for 12 weeks significantly reduced visceral fat area, body weight, and WC compared to a placebo group [24]. A cohort-based cross-sectional study showed that serum levels of ALA had a protective effect on body weight and were inversely associated with weight gain in children aged 5 to 12 years [25].

An experimental study by Adrina et al. showed that in the group fed with chia seeds as a rich source of ALA for three weeks, visceral fat tissue was significantly reduced compared to the control group.

Additionally, beneficial effects on blood lipids and glucose tolerance have been reported in the intervention group [33]. In contrast to the above studies, Australian and Spanish cross-sectional studies reported that plasma ALA concentration had a positive correlation with body fat and obesity [26][27]. To address the challenge of inconsistent study results, a study with a larger sample size and considering gender was needed.

Therefore, this study was designed with a larger sample size and separate analyzes for each gender. Also, in the case of the mentioned studies, supplementation [24] and plasma level [25][26][27] are considered the criteria for evaluating AL and ALA intake, but we assessed the dietary intake of ALA and LA. The following cellular mechanisms have been proposed to explain the relationship between higher intake of ALA and adiposity: a) ALA decreases body fat by stimulating the expression of hepatic fat oxidation and intestinal beta-oxidation genes [34][35]; b) ALA can inhibit the conversion of LA to arachidonic acid, which is a stimulator for adipogenesis through prostaglandin synthesis and CAMP activation [36]; c) by increasing the intake of dietary fatty acids, their tendency to accumulate increases. Among fatty acids, ALA has the highest tendency to oxidation and the lowest tendency to accumulate in humans [37].

In our study, a difference was observed between the males and females in the relationship between ALA dietary intake and adipose tissue. Different responses of body composition to intake of fatty acids in the gender have been observed in previous studies [38][39]. Men have been reported to have higher rates of fatty acid oxidation and lower resting energy expenditure than women [39]. In addition, the difference in sex hormones and adipose tissue percentage can affect the fats’ oxidation and storage [40].

The strength of the current study was the large sample size (3195 subjects) and using of an accurate method, and bioelectrical Impedance analysis, to evaluate body fat. Also, all required information was collected with validated questionnaires by trained experts to reduce any possible errors.

Additionally, because of a physiological difference between men and women in body fat percentage, the analysis was performed separately for both genders. Furthermore, to prevent the effect of energy intake, the dietary intake of ALA and AL was adjusted for total energy intake. However, there were some limitations in our study.

Firstly, the intake of ALA acid and LA included in our study was completely based on the consumption of meals, and the intake of possible supplements was not considered, which in some people could affect the daily intake of ALA and LA.

Secondly, factors including food preparation and cooking steps affecting the content of ALA and LA in foods have not been considered. Also, in this study, FFQ was used for assessing food intake.

FFQ is usually a tool for long-term evaluation of food intake, and estimating the amount of food consumed from an FFQ is not completely accurate, so measurement errors are always probable.

## Conclusion

Our findings show that a higher intake of ALA is associated with lower body fat in men. These results are important because, until now, all types of fatty acids have been equally accused of fat accumulation. Future studies on the quality of dietary fatty acids in weight management programs are suggested.

## Acknowledgments

The authors would like to thank all those who participated in this study. This study was supported by the Vice Chancellor of Research and Technology of Shiraz University of Medical Sciences, Shiraz, Iran. (grant No # 1399-01-84-22892)

## Conflict of Interest

We have declared that there is no conflict of interest.
